# rAAV production cost analysis: Indication-specific cost per dose and reduction strategies

**DOI:** 10.1038/s41434-026-00631-3

**Published:** 2026-07-21

**Authors:** Min Tae Park, Dan Matuszek, Angela Andaluz, Seyed Pouria Motevalian

**Affiliations:** Bioprocess Sciences, Pharma Services, Viral Vector Services, Thermo Fisher Scientific, Plainville, MA 02762 USA

**Keywords:** Gene therapy, Genetics

## Abstract

Recombinant adeno-associated virus (rAAV) vectors underpin many approved and late-stage gene therapies, yet manufacturing costs remain a major driver of therapy price. Here, we present the first comprehensive, platform-resolved cost analysis of rAAV production across three industrially relevant suspension platforms: transient transfection, baculovirus infection, and producer cell line (PCL). Using a bottom-up model, we decompose total cost-of-goods (COGs) into upstream, downstream, drug product, and quality control contributions at bioreactor scales from 50 L to 2000 L. We show that, on a per-batch basis, baculovirus infection is the most cost-efficient platform, followed by transient transfection and PCLs however, when costs are normalized to vector titer (cost per 1 × 10¹² vg), the ranking shifts, with transient transfection becoming the lowest-cost platform under the modeled assumptions, followed by baculovirus infection and PCL platforms, underscoring the dominant influence of productivity on unit cost. We identify platform-specific cost drivers—plasmids, transfection reagent, and media in transient transfection; media and perfusion consumables in PCL platforms; and affinity capture chromatography in baculovirus processes—with buffer preparation consistently representing the largest downstream material cost across platforms. We further quantify the impact of process development levers: transfection optimization and perfusion-based intensification reduce cost per dose by up to an order of magnitude, whereas affinity resin reuse and capsid enrichment strategies provide modest, incremental savings. Incorporating indication-specific annual viral genome demand reveals how process optimization and scale-up together reduce batch burden and lower dose cost by up to two orders of magnitude, suggesting the potential for improved supply feasibility, even for high-dose neuromuscular indications, under the modeled assumptions of productivity improvement and process optimization. Together, these results provide a quantitative framework linking platform choice, process scale, and unit operations to rAAV manufacturing COGs.

## Introduction

Recombinant adeno-associated virus (rAAV) vectors are the leading platform for in vivo gene delivery across a broad range of genetic and acquired diseases [1–3]. Despite impressive clinical progress, the high price of approved and emerging rAAV-based therapies has raised concerns about long-term affordability for patients, payers, and health systems [4, 5]. A substantial fraction of these prices has been attributed to manufacturing COGs, which remain high due to low volumetric productivity, expensive critical materials, and incomplete process standardization [6]. The challenge is particularly acute for indications requiring very high vector doses—such as systemic neuromuscular or neurological disorders—where dose requirements can reach 10^15^ vg per patient, and per-dose manufacturing cost may reach tens of thousands of US dollars even under efficient operation.

Industrial rAAV manufacturing has converged on three major suspension platforms: (i) triple-plasmid transient transfection in Human Embryonic Kidney (HEK) 293 cells, (ii) baculovirus infection of *Spodoptera frugiperda* clone 9 (Sf9) insect cells, and (iii) producer cell lines (PCLs) in mammalian hosts [7]. Transient transfection is highly flexible and rapid to deploy across serotypes, but its dependence on large quantities of plasmids and transfection reagent imposes high upstream process (USP) material costs [6, 8, 9]. Baculovirus infection in Sf9 cells offers lower media cost and simplified upstream operations at large scale, but requires generation and control of recombinant baculovirus stocks and increased attention to viral and host-cell impurities during downstream process (DSP) [10–12]. PCL platforms promise streamlined production without repeated plasmid delivery, yet demand lengthy cell line development, serotype-specific optimization, and often rely on perfusion, which substantially increases media and single-use consumable usage [13, 14].

Although the technical attributes of these platforms have been widely discussed, quantitative, platform-level cost benchmarking remains scarce. Existing studies have focused mainly on technical performance or have treated “rAAV manufacturing” as a single modality, without resolving platform-specific cost structures or including newer baculovirus and PCL platforms [8, 12, 13]. Critically, there is little published guidance on (i) how total and per-dose costs partition across USP, DSP, drug product (DP), and quality control (QC) activities for each platform; (ii) how these costs evolve with production scale; and (iii) which concrete process development strategies provide the largest, quantifiable reductions in cost per vg or per dose. This lack of transparency hampers rational platform selection and process design, particularly when aligning manufacturing strategy with indication-specific dose requirements and patient population size.

In this study, we address these gaps by developing a detailed, bottom-up cost model for rAAV production in transient transfection, baculovirus, and PCL platforms operated under GMP conditions. We (i) generate platform-specific cost breakdowns across USP, DSP, DP, and QC; (ii) further decompose costs into raw materials and conversion components, including operations, facility, and release testing, (iii) compare cost per batch, per 10^12^ viral genomes (vg), and per dose at bioreactor scales from 50 L to 2000 L; and (iv) quantify economies of scale. We further evaluate major process development levers, including transfection optimization, upstream perfusion-based intensification, affinity resin reuse, and capsid enrichment in anion-exchange (AEX) polishing, and quantify their impact on COGs at 2000 L scale. Finally, we estimate clinical indication-specific annual vg demand and cost per dose, and quantify reductions achieved through process optimization and intensification.

To our knowledge, this is the first study to provide a platform-resolved, quantitative cost dissection of rAAV manufacturing together with a set of quantitatively evaluated, numerically grounded cost-reduction strategies, including cost-per-dose estimates for major clinical indications where rAAV is commonly used. The resulting framework is intended to support evidence-based platform selection and process development decisions aimed at improving the economic sustainability and accessibility of rAAV gene therapies. This analysis is limited to manufacturing costs and does not include broader cost components such as discovery, clinical trials, or regulatory approval.

## Materials and methods

### Overall cost model framework

**Fig. 1:**
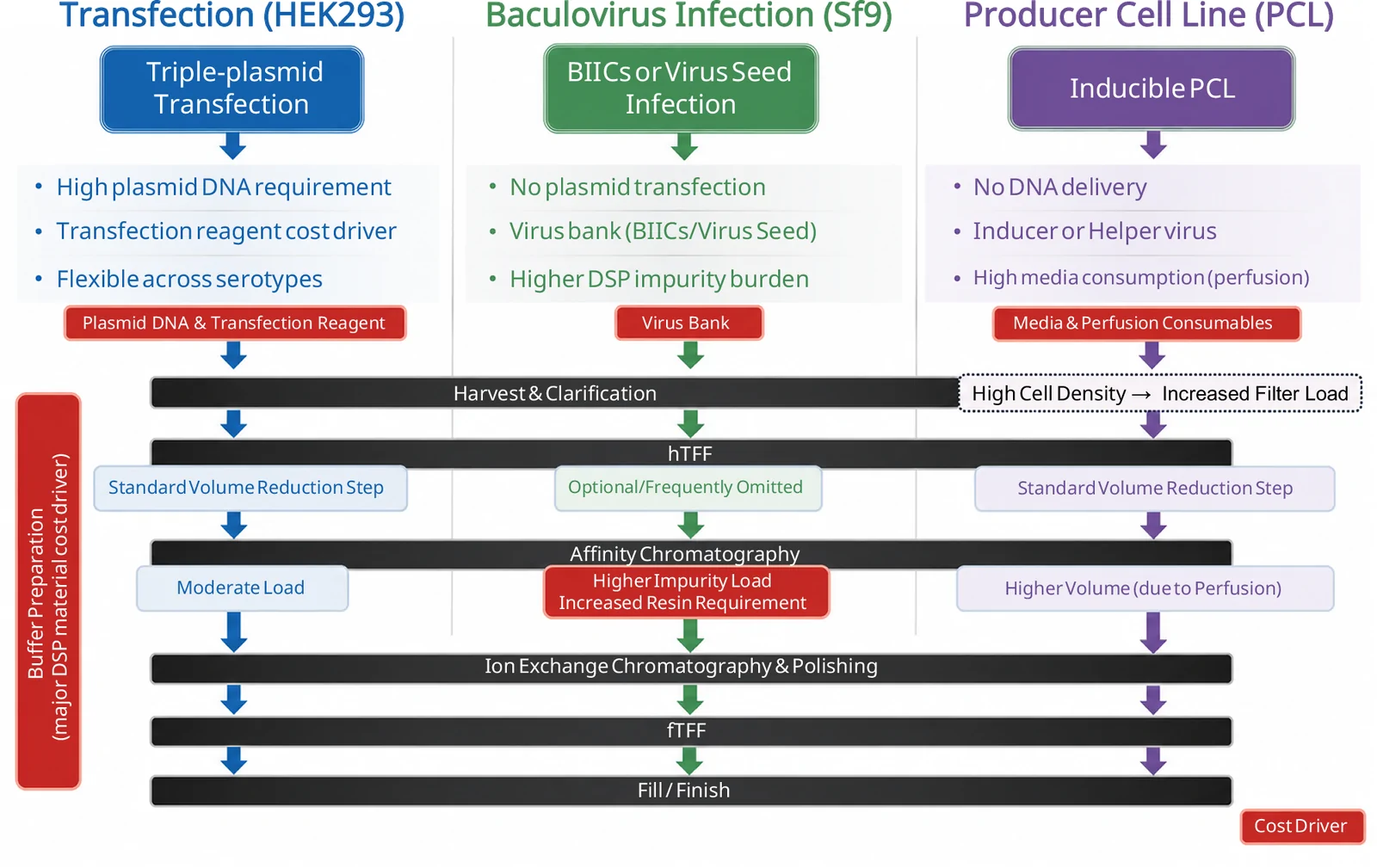
Platform-specific rAAV manufacturing workflows and associated cost drivers. Transient transfection relies on plasmids and transfection reagent, baculovirus infection shifts costs toward DSP because of a higher impurity burden, and PCL platforms are characterized by perfusion-based operation and elevated media consumption. Major cost-driving steps are highlighted for each platform.

A comprehensive, bottom-up cost model was developed to compare rAAV manufacturing across three suspension platforms ─ transient transfection, baculovirus infection, and producer cell line (PCL) ─ as described by Park et al. [7]. Figure 1 illustrates the typical rAAV production workflow, highlighting key stages such as cell expansion, virus production, harvest, and purification. In USP, host cells such as HEK293, Sf9, or HeLa are expanded and then transfected, infected, or induced to produce rAAV vectors, depending on the platform [7, 15, 16]. The DSP includes harvest, clarification, and purification steps required to achieve the desired product quality attributes [7, 16].

This analysis represents a baseline scenario and does not explicitly incorporate manufacturing variability such as batch failures, contamination events, or QC-related rejection. In practice, such factors can reduce the number of successful batches and increase the effective cost per dose. Although a generalized treatment of manufacturing variability is challenging due to limited publicly available data, future work applying probabilistic approaches or facility-specific modeling could further enhance the robustness of the analysis.

To improve transparency, key modeling assumptions are explicitly summarized. Platform-specific productivity values (vg titers) were derived from a combination of internal experimental data and literature-reported ranges and are intended to represent achievable, but not universal, scenarios that may vary depending on process maturity, serotype, and facility-specific implementation [7]. Downstream recovery was treated as a fixed overall value based on representative process data, and variability across scales or platforms was not explicitly modeled. The model further assumes consistent process performance without batch-to-batch variability and applies regionally normalized cost assumptions without incorporating geographic differences in labor, facility, or supply chain costs. Due to limited publicly available data, a full probabilistic uncertainty analysis was not performed; however, these assumptions represent key sources of uncertainty and should be considered when interpreting the results.

### Cost categories and definitions

The total cost of manufacturing was divided into direct material (raw material) costs and conversion costs. Direct costs for USP include platform-specific media and supplements; plasmids and transfection reagent for transfection platform; Baculovirus-Infected Insect Cells (BIICs) or virus seed stocks for baculovirus infection platform, and perfusion hardware and consumables such as ATF filters for PCL. Direct costs for DSP include clarification filters, tangential flow filtration (TFF) filters and flow path, affinity and anion-exchange resins and columns, buffers, and single-use manifolds and bags. DP materials consist of formulation materials, vials, sterile filters, and fill/finish disposables, while QC direct costs primarily comprise supplies used for lot release testing.

Conversion costs consist of direct labor and indirect expenses, as detailed in Table S1 (see the Supplementary Information – Section 1: Conversion Cost Model). Direct labor includes USP, DSP, DP, QC, and Quality Assurance (QA) personnel, along with the management and supervision responsible for overseeing these teams. Indirect expenses consist of facility capital costs, maintenance, local taxes, insurance, depreciation (facilities and equipment), and general utilities. While the conversion cost model was applied consistently across scales, DP and QC conversion costs were applied per lot and did not scale with batch size, increasing their share of total cost at smaller scales.

### Platform-specific development costs

The cost model also accounted for the expenses associated with generating the master BIICs (MBIICs) for the baculovirus infection platform, as well as the costs associated with producer cell line development and helper virus bank establishment for the PCL platform. Further details are provided in Table S2 (see Supplementary Information, Section 2: PCL and BIICs Development Costs and Allocation to Manufacturing).

### Scale-dependent material cost analysis

The impact of production scale on material cost efficiency within a representative transient transfection process was evaluated by modeling batch sizes of 50 L, 250 L, 1000 L, and 2000 L. The analysis encompassed USP materials (single-use bioreactors, sterilizing-grade filters, single-use bags, media, preparation buffers, plasmids, transfection reagent, and endonuclease), DSP materials (clarification depth filters, sterilizing-grade filters, virus-retentive filters, TFF cassettes, hollow-fiber modules, and chromatography resins and columns for affinity and polishing chromatography steps), as well as DP and QC consumables. For a complete list of DSP unit operations and their associated recoveries, refer to the Supplementary Information (Section 3: Downstream Processing - Steps and Recoveries).

### Process development and intensification modeling

Details of the USP and DSP development and process intensification efforts aimed at cost reduction are provided in the Supplementary Information (Section 4: Transfection Process Optimization, Section 5. Upstream Process Intensification via Perfusion, and Section 6: Percent Full Capsid Enrichment via AEX). The cost model described above was then applied to quantify the resulting cost reductions achieved when the optimized process is implemented at the 2000 L scale. The productivity improvements from design-of-experiment (DOE) optimization and perfusion-based intensification were derived from a combination of internal experimental data and literature-reported ranges, as referenced in the corresponding sections. These values were implemented in the model as representative scenarios rather than universally achievable outcomes. Unless otherwise stated, all cost calculations represent a baseline (idealized) scenario, assuming successful batch execution without process failure, batch rejection, or quality-related loss.

### Cost-per-dose modeling and patient population analysis

For cost-per-dose analyses, eligible patient populations for AAV gene therapy were estimated based on disease prevalence per 100k individuals. This approach captures the total living patient population, including both newly diagnosed and existing cases worldwide. Given a prevalence of *N* cases per 100k individuals, the affected population was extrapolated using regional demographic data. All existing patients were assumed to be treated over a 10-year horizon, and newborn incidence was incorporated annually (assuming ~70 million global births per year). Based on these assumptions, the annual number of treated patients was derived from the total estimated patient population. Adjustments were then applied for diagnostic rates and access to genetic testing (e.g., ~50% in North America and Europe, ~30% in Asia, and ~20% in the rest of the world). Finally, clinical eligibility criteria—such as age and disease stage—were applied to refine the treatable patient pool per year. This annual eligible population, together with indication-specific dose requirements per patient, was used to calculate the total annual vg demand and subsequently, the number of batches per year and cost per dose. For full details, refer to Supplementary Information (Section 7: Indication-Specific Patient Population, Dose, and rAAV Demand).

## Results and discussion

### Overall manufacturing costs across production platforms

**Fig. 2:**
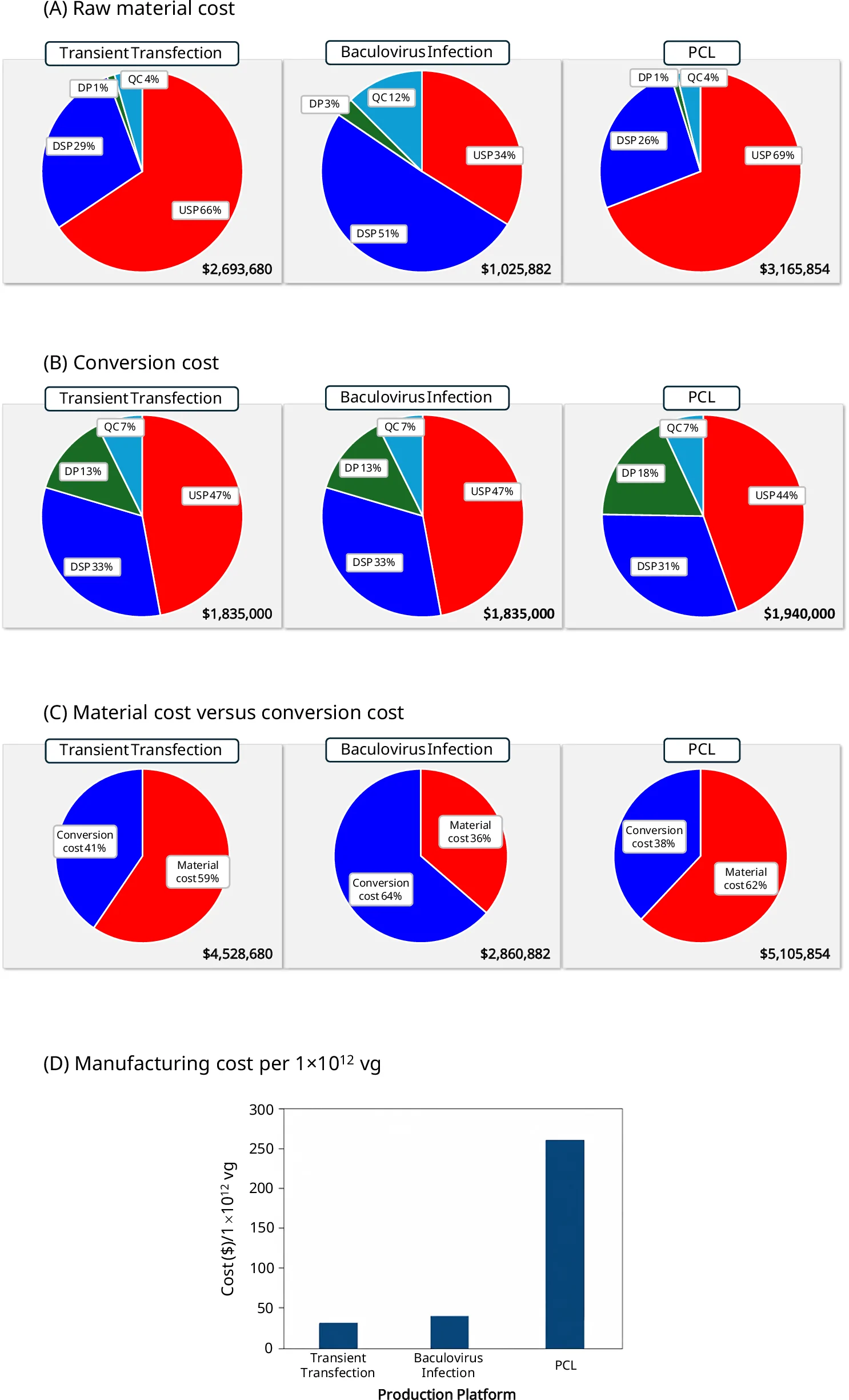
Comparison of total rAAV manufacturing costs across production platforms at the 2000 L scale. On a total manufacturing cost basis, the PCL platform incurred the highest cost, followed by transient transfection, whereas the baculovirus infection platform was the most cost-eff ective. When normalized per 1×10¹² vg, the ranking shifted, underscoring the impact of process productivity on unit cost. **A** Raw material cost by process stage (USP, DSP, DP, QC). **B** Conversion cost by process stage. **C** Material cost versus conversion cost. **D** Manufacturing cost per 1×10¹² vg. Panels **A**–**C** are shown for each platform with totals below each chart.

The PCL platform exhibits the highest manufacturing costs, followed by the transient transfection platform, while the baculovirus infection platform represents the most cost-effective option (Fig. 2). When normalized by vg titer, however, the ranking changes: at the 2000 L scale, the cost per 1 × 10^12^ vg for rAAV production is $260.8 for the PCL platform, $38.6 for the baculovirus infection platform, and $30.8 for the transient transfection platform (Fig. 2D). This shift reflects the influence of process productivity—particularly vg titer—on unit cost and underscores why cross-platform comparisons depend heavily on productivity assumptions. For example, a recent case study reported ~10-fold higher upstream titers and >10-fold cost reductions when moving from HEK293 transfection to an Sf9-baculovirus platform [15, 17]. Separately, the current PCL platform typically delivers ~2 orders of magnitude lower vg titers than transient transfection or baculovirus infection platforms, translating into a higher cost per vg consistent with the trend observed in our study [18]. Although transient transfection offers flexibility, it remains less economical compared to the baculovirus infection platform for large-scale manufacturing because of high USP material costs, particularly plasmids and transfection reagent. The baculovirus infection platform delivers significant cost advantages at large-scale production but incurs additional costs for baculovirus generation and banking (approximately 2.6% of total manufacturing cost per batch). PCL platform, despite its high initial development costs and royalty payments (approximately 0.9% of total batch cost), offers long-term cost advantages if sufficient improvements in productivity can be achieved. In addition, although current implementations often rely on perfusion, alternative operating modes (e.g., fed-batch or hybrid strategies) could further improve cost efficiency by reducing media consumption, potentially resulting in cost structures more comparable to baculovirus-based platforms. However, such scenarios remain to be experimentally validated and should be interpreted as forward-looking considerations.

Figure 2 summarizes total manufacturing costs across the major production platforms at the 2000 L scale. USP costs exceeded DSP costs for both the transient transfection and PCL platforms. Culture media costs (a considerable contributor to raw material cost) are substantially higher in these platforms compared to the baculovirus infection platform. The difference arises primarily from (i) the need for supplemental nutrients such as glutamine and glucose during production in transfection and PCL platforms - glutamine being essential for mammalian cells but not required for insect cells in the baculovirus/Sf9 platform—and (ii) the generally lower unit cost of insect cell media relative to mammalian cell media, resulting in reduced USP cost burdens for the baculovirus/Sf9 platform. For the transfection platform, USP costs significantly exceeded those of DSP, DP, and QC operations, primarily due to the additional major expenses associated with plasmids, transfection reagent, and optional boosters required in this platform. Notably, in the baculovirus/Sf9 platform, DSP processing costs exceeded USP costs because the baculovirus process omits costly transfection and perfusion-related raw materials, thereby yielding lower USP expenses relative to the other platforms.

Conversion cost breakdowns across USP, DSP, DP, and QC were relatively similar for all three platforms. This similarity primarily reflects the identical operating scale used across platforms, which yielded comparable allocations of conversion costs.

While raw materials and consumables meaningfully contribute to overall manufacturing costs in biologics manufacturing at CMOs, conversion costs—facility, labor, and overhead—often dominate at large scale [19]. In our analysis, this pattern was observed only for the baculovirus/Sf9 platform. For the transfection and PCL platforms, substantial direct-material expenditures associated with transient transfection (e.g., plasmids, transfection reagent, optional enhancers) and perfusion operations (e.g., ATF hardware and consumables) shifted the balance, making direct materials—not conversion costs—the primary cost drivers.

### Comparative cost analysis of upstream materials

**Fig. 3:**
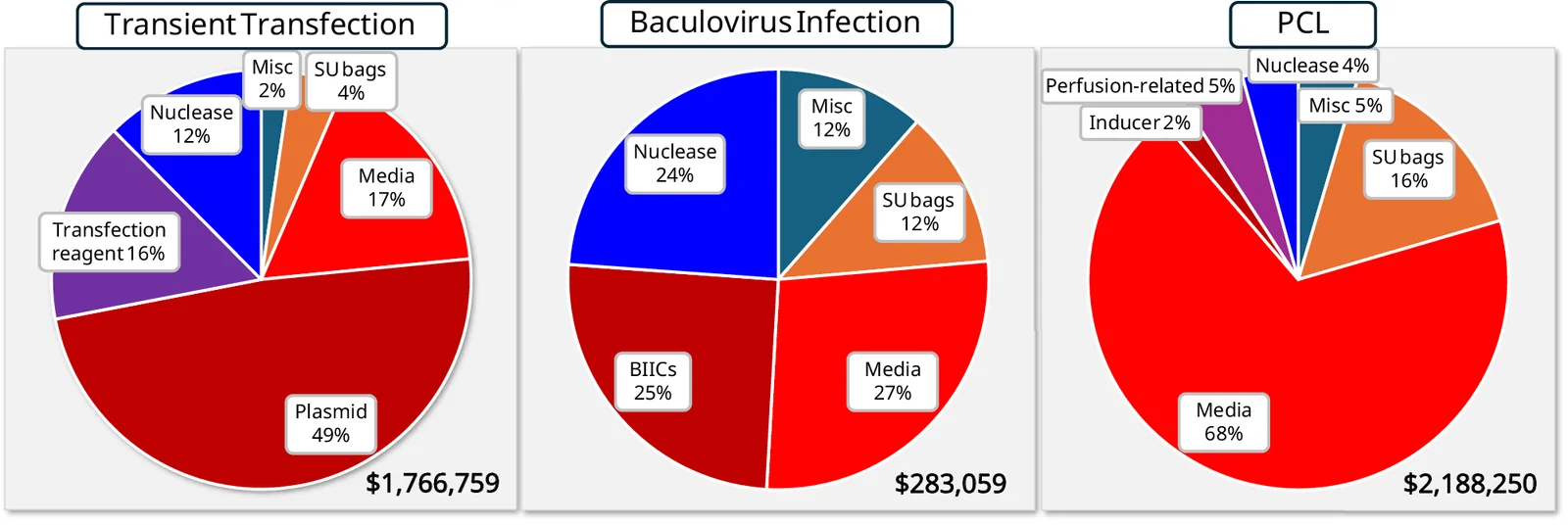
Comparisn of upstream material costs among the three platforms. The transient transfection platform is primarily driven by plasmids, media, and transfection reagent, whereas the baculovirus infection platform shows a more balanced cost distribution. The PCL perfusion-based platform is dominated by media costs because of the high media consumption associated with perfusion operation.

Figure 3 illustrates the distribution of USP raw material costs across the three major rAAV production platforms. Overall, USP raw material costs are 7.7-fold and 6.2-fold higher in the PCL and transfection platforms, respectively, compared with the baculovirus infection platform at the 2000 L scale. In the transient transfection platform, the major cost components include plasmids, media, transfection reagent, and Benzonase, which together account for more than 94% of the overall USP material cost. Additional cost contributors include SU bags and miscellaneous items such as filters and manifolds. In contrast, the baculovirus infection platform has costs more evenly distributed among various categories. Although the primary cost drivers are media, Benzonase, and BIICs, which together account for 76% of the overall USP material cost, additional expenses such as SU consumables and miscellaneous items are relatively evenly distributed. The PCL perfusion-based platform shows a skewed USP cost distribution dominated by media, reflecting the high consumption inherent to perfusion. Media alone accounts for 68% of the USP material cost, while SU bags and alternating tangential flow (ATF) filters together contribute approximately 21%. In contrast to the other two platforms, the PCL perfusion platform exhibited a relatively lower endonuclease (e.g., Benzonase) cost ratio due to the high media cost. Conversely, the share of single-use bag costs was higher than in the other platforms, reflecting the consumable intensity of perfusion. Because endonuclease is a notable cost burden in rAAV manufacturing, perfusion can be an effective lever for material cost reduction—provided it delivers sufficient productivity to offset the added single-use and perfusion-related expenses.

### Comparative cost analysis of downstream materials

**Fig. 4:**
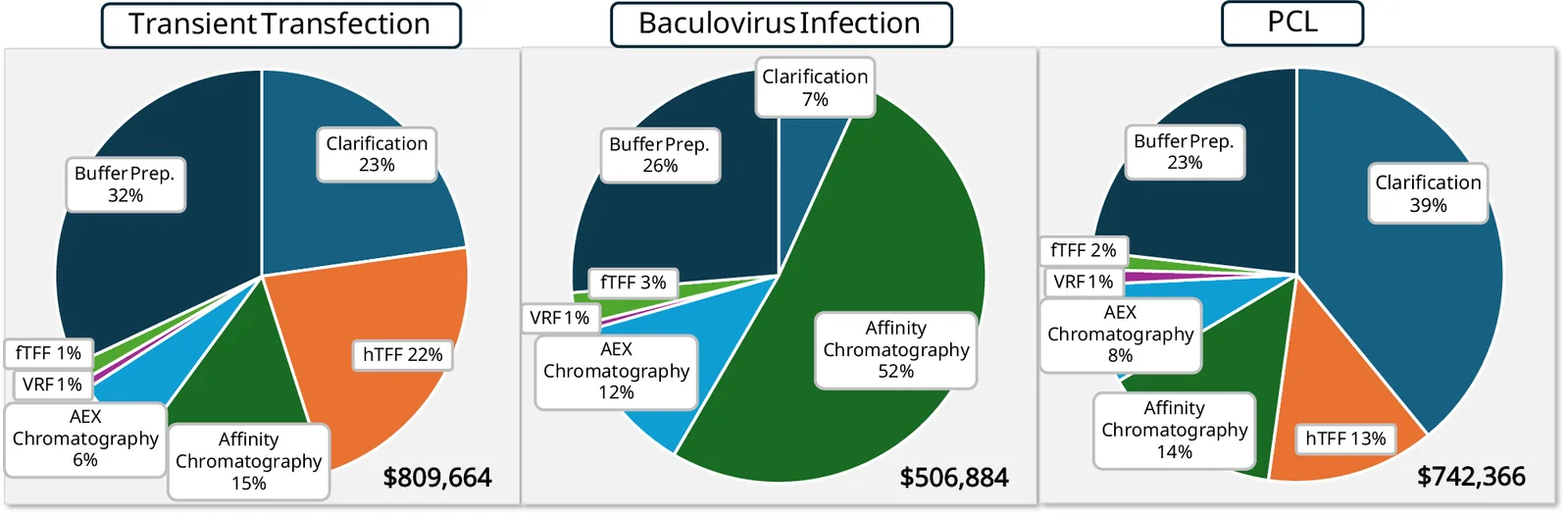
Comparison of downstream material costs among the three platforms. The transient transfection platform incurs the highest DSP material cost ($809 k), followed by the PCL platform ($724 k) and the baculovirus infection platform ($506 k). Buffer preparation is a major cost contributor across all platforms, while clarification and affinity chromatography are key cost drivers in the PCL and baculovirus infection platforms, respectively.

Figure 4 illustrates the distribution of downstream raw material costs across the three production platforms at the 2000 L production scale. Overall, each platform follows a similar downstream sequence: clarification, harvest tangential flow filtration (hTFF) (except for the baculovirus infection platform), affinity chromatography, AEX chromatography, viral reductive filtration (VRF), and final TFF. Buffer preparation represents 23–32% of total downstream raw material costs across all platforms. It is the largest cost driver in transient transfection platform and still a major contributor in the other platforms. The cost of water for injection (WFI) was negligible and thus excluded from all buffer preparation calculations. Clarification, hTFF, and affinity capture follow as secondary cost drivers, with their relative importance and associated costs differing by platform.

Clarification costs are largely driven by the scale and complexity of the feed stream, with filter area and depth filter usage increasing sharply for high-cell-density or lysed harvests. For hTFF, costs stem primarily from the use of large membrane surface areas and single-use cassettes, which scale directly with process volume.

The percentage contribution of affinity chromatography varies substantially between platforms. In the baculovirus infection platform evaluated here, which lacks an hTFF step, the affinity column was oversized to enable direct capture of upstream material without prior volume reduction. This configuration required larger resin volumes to avoid excessively long loading times, thereby increasing material cost. In addition, impurities such as host-cell DNA and protein, which are partially removed during hTFF in other platforms, remain present in the baculovirus harvest. These impurities typically increase viscosity and can foul the resin, effectively reducing available binding capacity and capture efficiency. Similar observations have been reported for protein A chromatography in mAb production [20, 21]. Together, these process-specific factors explain why affinity costs for the baculovirus infection platform are markedly higher than in the other platforms.

AEX chromatography is relatively inexpensive across platforms, contributing only 6–12% of total downstream material costs. Finally, VRF and final TFF each account for a small fraction of downstream raw material costs (less than 5%).

### Manufacturing cost reduction strategies

#### Scale-up and cost efficiency

**Fig. 5:**
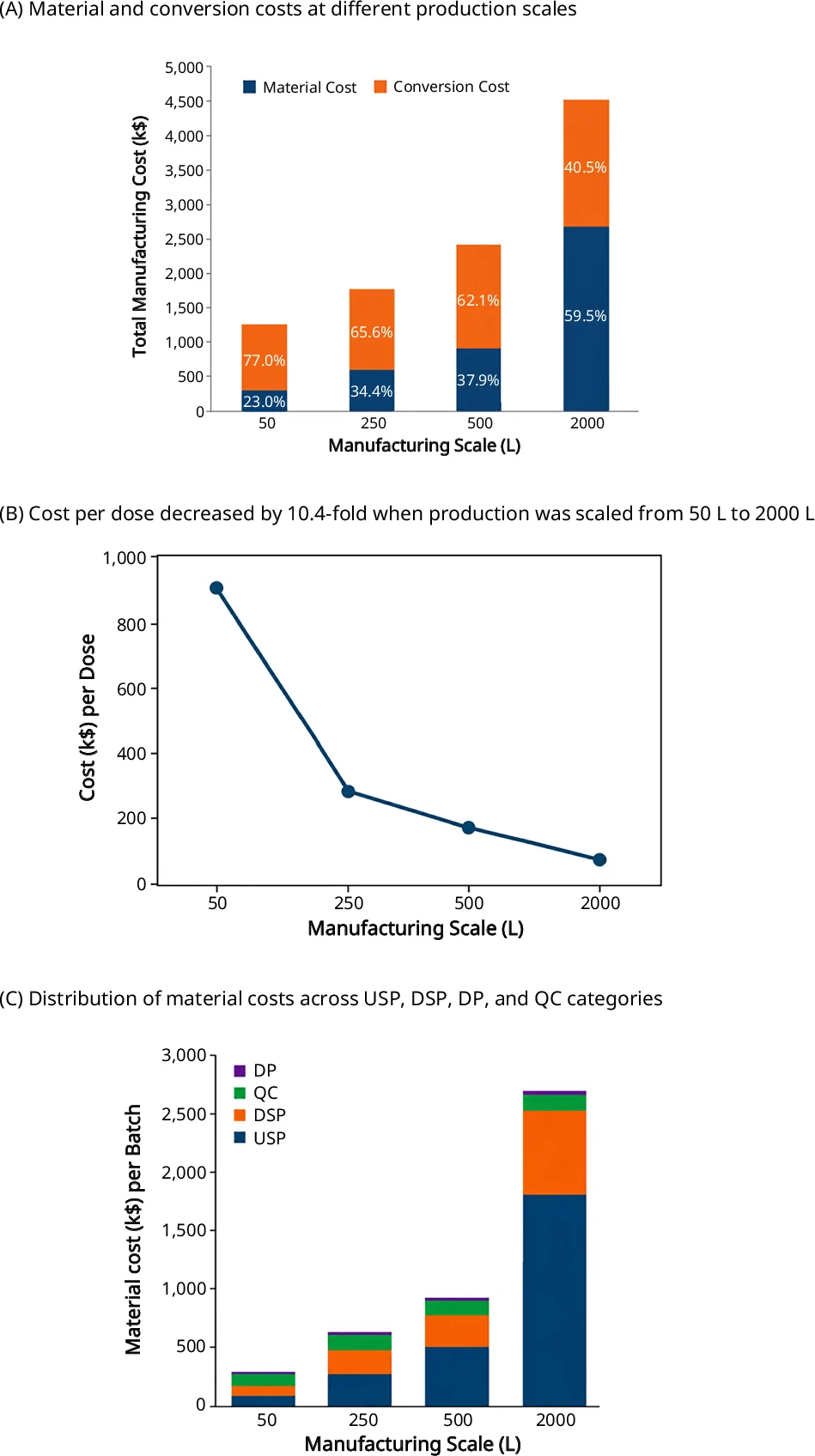
Scale-dependent rAAV production costs and dose economics in the transient transfection platform. Costs were modeled for a transient transfection rAAV process at four scales (50, 250, 500, 2000 L), revealing that larger scales markedly improve cost effi ciency. **A** Total manufacturing cost per batch as stacked bars split into material (dark blue) and conversion (orange) cost, with segment labels showing each share. As scale increases, fixed conversion costs are diluted and the material share rises shifting the cost structure toward consumables. **B** Cost per dose, which fell 10.4-fold from 50 L to 2000 L, the dominant driver of improved dose economics. **C** Material cost per batch split across upstream (USP, dark blue), downstream (DSP, orange), quality control (QC, green), and drug product (DP, purple); USP remains the largest material-cost component at every scale, marking upstream consumables as the key target for further cost reduction.

Scale-up significantly reduces total manufacturing cost for rAAV-based therapeutics. When the transient transfection process is scaled from 50 L to 2000 L—a 40-fold increase in production volume at a constant USP titer of 5.0 × 10¹¹ vg/mL and an overall DSP recovery of 23% as reported in the Supplementary Information (Table S3)—the total cost increases from $1.24 M to $4.53 M, representing only a 3.56-fold increase (Fig. 5A). Additionally, the ratio of conversion to material cost decreases sharply with scale, from about 3.35 at 50 L to 0.68 at 2000 L. This reflects that conversion costs—driven by labor, facility overhead, and other fixed operational expenses—do not scale linearly with production volume and become diluted at larger scales. In contrast, material costs such as plasmids, transfection reagent, and chromatography resins scale proportionally with batch size, leading to a lower conversion-to-material cost ratio at higher manufacturing scales.

As shown in Fig. 5B, scaling up production leads to a substantial reduction in cost per dose. For the transient transfection platform described earlier and assuming Duchenne muscular dystrophy as the clinical indication (2.8 × 10¹⁵ vg/dose), the cost per dose decreases from $892k at 50 L to $86k at 2000 L, representing an overall cost reduction of 10.4-fold. Similarly, the cost per 1 × 10¹² vg at BDS (Bulk Drug Substance) decreases sharply from $320 at 50 L to $31 at 2000 L, demonstrating pronounced economies of scale across the transient transfection platform.

Fig. 5C shows the variation in material cost across different manufacturing scales for rAAV production using the transient transfection process. The total material cost per batch increases markedly with scale, rising from approximately $0.29 M at 50 L to $2.7 M at 2000 L. The cost structure also evolves as the process scales up. At the 50 L scale, USP, DSP, QC, and DP contribute roughly 28%, 26%, 42%, and 4% of the total cost, respectively. With increasing scale, USP material cost becomes progressively dominant—accounting for approximately 45% at 250 L, 56% at 500 L, and 66% at 2000 L. In contrast, the proportional contributions of DSP, QC, and DP decrease to 29%, 4%, and 1%, respectively, at 2000 L. These results indicate that while total material costs rise with scale, the increase is largely driven by USP raw materials and consumables, reflecting their sensitivity to reactor volume and production scale. DSP and QC components grow more modestly, highlighting improved material-use efficiency at larger scales.

Another advantage of scale-up is that a smaller fraction of the total vial count is consumed by QC release testing. Because 20–40 vials are typically allocated for release, this fixed requirement represents a much smaller proportion of larger-scale batches, thereby increasing the amount of material available for patient dosing [22, 23].

#### Process development and optimization

USP and DSP process development and optimization—whether aimed at increasing USP productivity, reducing raw material consumption, or intensifying unit operations—can substantially lower the overall cost per dose. Here we identify key areas of process optimization that drive cost efficiency and quantitatively assess the resulting savings when scaled to 2000 L manufacturing. For USP development, two optimization scenarios were evaluated. The first involved a two-step optimization of the transient transfection process using a Quality-by-Design Design-of-Experiment (DOE) approach. The second focused on process intensification through implementation of a perfusion-based USP strategy. In DSP, we quantified cost savings associated with resin reuse and further demonstrated that optimization of the AEX chromatography step can increase the proportion of full capsids without significantly affecting step yield, hence the batch cost.

#### Transfection process optimization

In the first case study, the transient transfection process was optimized by adjusting the total DNA amount, plasmid ratios, and viable cell density at the time of transfection. The vg titers at harvest for the benchmark, DOE1-optimized, and DOE1 + DOE2-optimized processes were 6.0 × 10¹⁰ vg/mL, 2.9 × 10¹¹ vg/mL, and 8.8 × 10¹¹ vg/mL, respectively. Details of the optimization can be found in the Supplementary Information (Section 4: Transfection Process Optimization). Assuming scale-up of this process to a 2000 L bioreactor for a neurodegenerative disease requiring 9.1 × 10^13^ vg/kg of patient weight (2.8 × 10¹⁵ vg/dose), the benchmark, DOE1-optimized, and DOE1 + DOE2-optimized processes would yield sufficient material to treat 8, 38, and 116 patients, respectively—representing more than a 14-fold increase in patient coverage.

**Fig. 6:**
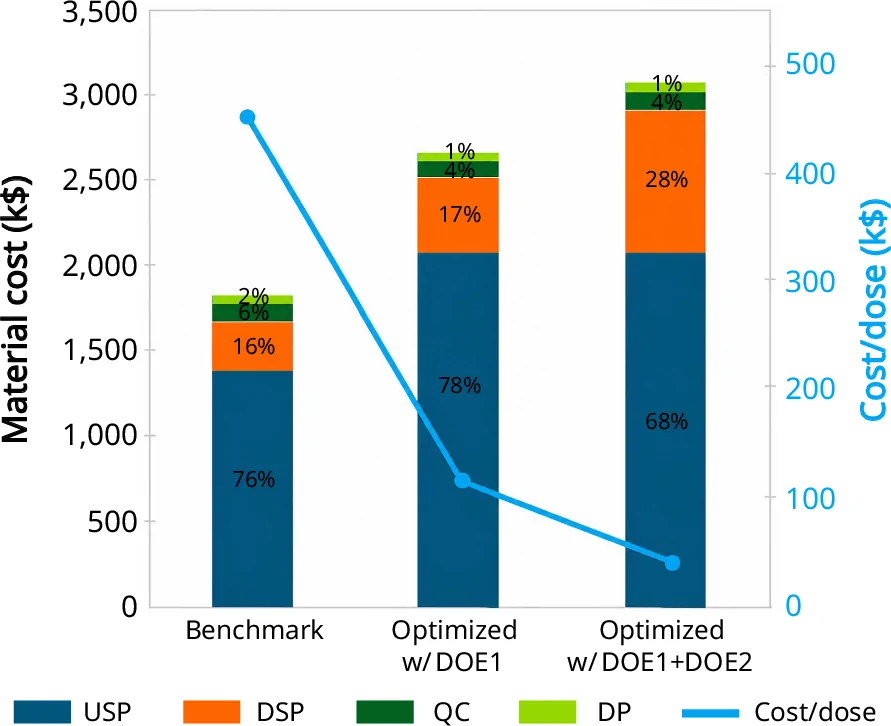
Cost benefits of rAAV transfection optimization. Optimization of the triple-plasmid transfection process substantially increased vg titers, resulting in an approximately 14-fold increase in patient coverage. Despite higher material costs for the DOE-optimized processes, the overall cost per dose was substantially reduced, demonstrating improved process efficiency and scalability.

This substantial gain in productivity was accompanied by an increase in total material cost for the DOE-optimized processes. As shown in Fig. 6, the DOE1- and DOE1 + DOE2-optimized processes incurred 47% and 69% higher material costs, respectively, when scaled to 2000 L production. This increase was due to higher DSP purification costs resulting from improved USP titers in both DOE1 and DOE2 optimizations. Additionally, USP material costs rose by approximately 50% in the DOE1-optimized process due to increased DNA and transfection reagent usage. Since DOE2 optimization focused solely on plasmid ratio adjustment, its total USP cost remained largely unchanged at approximately $2.1 M. Despite these increases in material cost, the overall cost per dose was reduced by ~74% (from $458k to $117k) and ~91% (from $458k to $42k) for the DOE1- and DOE1 + DOE2-optimized processes, respectively.

Changes in vg titer can be accompanied by shifts in the full-to-empty capsid ratio, which may impact downstream purification efficiency and overall recovery. Therefore, it is critical to optimize % full capsid during USP to avoid placing additional burden on downstream purification steps through the need to remove empty and partial capsids, which can negatively affect yield.

#### USP intensification via perfusion

While the majority of rAAV USP production processes rely on fed-batch operation, process intensification through perfusion can further enhance productivity, reduce process footprint, and provide significant economic advantages. In this case study, a benchmark fed-batch process was converted to a perfusion-based rAAV production process. Two configurations were evaluated: the first employing perfusion only prior to transfection, and the second incorporating perfusion both before and after transfection. The vg titers at harvest for the perfusion-pre-transfection and perfusion-pre-and-post-transfection processes were 3.4 × 10¹¹ vg/mL, and 9.4 × 10¹¹ vg/mL, respectively. Details of the perfusion strategies are provided in the Supplementary Information (Section 5: USP Intensification via Perfusion). Assuming scale-up to a 2000 L bioreactor for a neurodegenerative disease requiring 9.1 × 10^13^ vg/kg of patient weight (2.8 × 10¹⁵ vg/dose), the benchmark, perfusion-pre-transfection, and perfusion-pre-and-post-transfection processes would yield sufficient material to treat 8, 45, and 123 patients, respectively—representing a more than 15-fold increase in patient coverage.

**Fig. 7:**
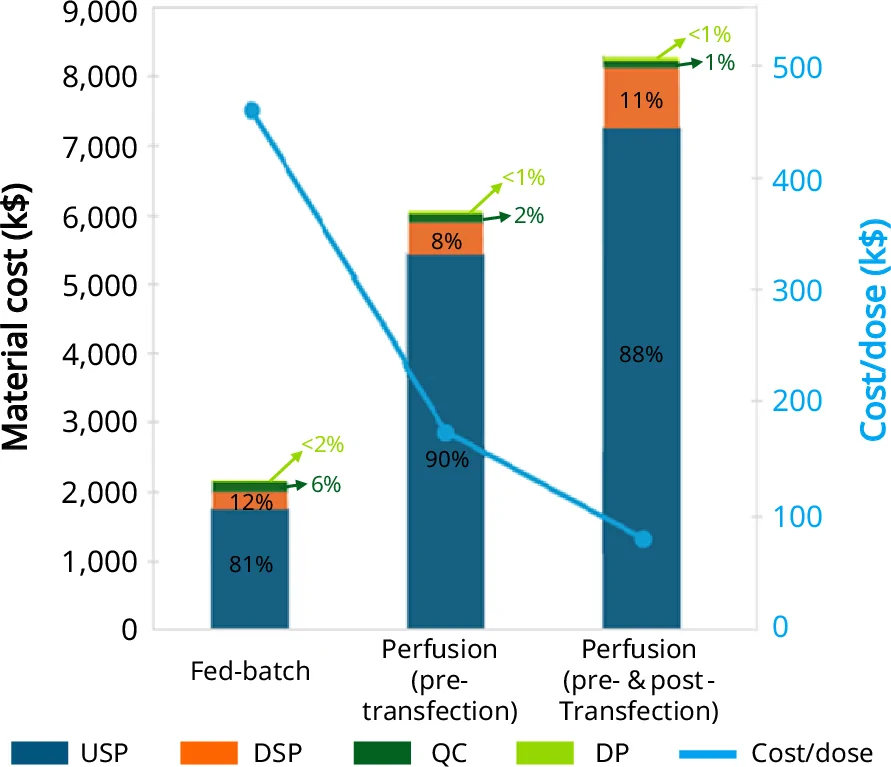
Cost reduction via process intensification. Converting a fed-batch process to perfusion-based rAAV production significantly increased vg titers, resulting in a more than 15-fold increase in patient coverage. Despite higher material costs for the perfusion processes, the overall cost per dose was dramatically reduced, demonstrating the economic benefits of process intensification.

The increased productivity achieved through perfusion was accompanied by higher total raw material costs. As shown in Fig. 7, the perfusion-pre-transfection and perfusion-pre-and-post-transfection processes incurred 333% and 456% higher material costs, respectively, when scaled to 2000 L production. This increase was primarily driven by the additional media, plasmids, and transfection reagent required for perfusion-based operation, as well as elevated DSP purification costs associated with improved USP titers. Despite these increases in material cost, the overall cost per dose—assuming production at the 2000 L scale for the neurodegenerative disease model described earlier—was reduced by approximately 62% (from $458k to $176k) and 82% (from $458k to $82k) for the perfusion-pre-transfection and perfusion-pre-and-post-transfection processes, respectively.

## Downstream affinity resin reuse

Resin reuse is one of the well-established cost reduction strategies commonly employed in more mature modalities such as antibody production [24]. To evaluate the economic impact of resin reuse for rAAV manufacturing, we distributed the cost of the POROS™ CaptureSelect™ AAVX resin across multiple reuse cycles while holding load density constant. This approach assumes stable dynamic binding capacity and column performance across cycles, consistent with reported reuse data. The single-use affinity unit operation was $122k, comprising $30k in consumables and $91k in resin- or column-related expenses.

**Fig. 8:**
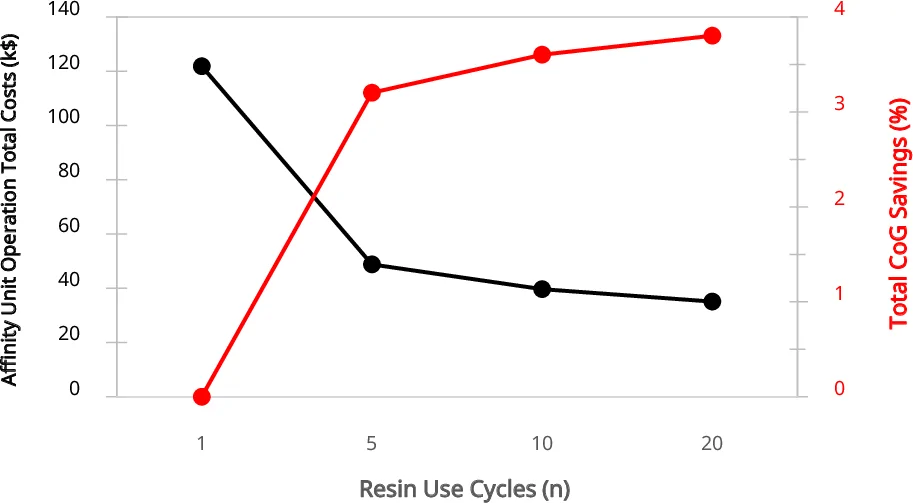
Impact of POROS™ CaptureSelect™ AAVX resin reuse on 2000 L process economics. Distributing the resin cost across multiple reuse cycles (1, 5, 10, 20) reduced the affinity step cost by up to 71% in the theoretical 20-cycle scenario and lowered total material cost by ~3–4%. Most savings were realized within the first 5–10 cycles, with diminishing returns beyond 10 cycles.

As shown in Fig. 8, reuse over 5 cycles reduced the affinity step material cost by approximately 60%, from $122k to $48.8k, saving $73.1k per batch and lowering total material cost from $2.28 M to $2.21 M ( ~ 3.2%). At 10 and 20 reuse cycles, total material cost savings increased modestly to 3.6% and 3.8%, respectively. These estimates assume consistent product yield and impurity clearance across reuse cycles; deviations in either parameter would reduce the realized savings. While reuse up to 10 cycles is supported by recently published data from the supplier demonstrating stable performance under sodium hydroxide Cleaning-in-Place (CIP) conditions [25], the 20-cycle scenario represents a theoretical upper bound intended to illustrate the diminishing returns of extended reuse.

This analysis reflects resin cost distribution only and excludes additional operational requirements associated with reuse, including additional raw materials/reagents, operator time, analytical testing, and validation activities, which scale with the number of cycles and would further reduce net savings. Furthermore, demand for DP may be limited to one production run per year (e.g., the retinal dystrophy indication shown in Table 1), which may constrain the practical ability to fully realize reuse lifetimes due to resin storage limits or campaign scheduling. Overall, while resin reuse reduces affinity step costs, its impact on total manufacturing cost at the 2000 L scale remains limited to the low single-digit range.

**Table 1:** Indication-based comparison of rAAV manufacturing costs depending on USP development.

A Indication-specific prevalence, doserequirements, and global rAAV demand [29].				
Indication	Prevalence^1^	Patients worldwide^2^	Dose (vg) per patient	Total demand (vg) worldwide per year^3^
RPE65 Mutation-associated Retinal Dystrophy	0.25 per 100k	4,921	1.5×10^11^	8.0×10^13^
Age-related Macular Degeneration (AMD)	350 per 100k	42,019,200	3.3×10^11^	9.2×10^17^
Hemophilia B	3.8 per 100k^4^	37,217	1.4×10^15^	5.7×10^18^
Spinal Muscular Atrophy (SMA)	1 per 100k	8857	1.7×10^15^	2.8×10^18^
Duchenne Muscular Dystrophy (DMD)	7.1 per 100k^d^	54,881	2.8×10^15^	1.7×10^19^
Hemophilia A	17.1 per 100k^d^	167,479	4.2×10^15^	7.6×10^19^

B Number of batches required to meet worldwide patient demand across the evaluated indications at 50 L and 2000 L production scales under benchmark, upstream-optimized (DOE1 + DOE2), and intensified USP scenarios.						
Indication	Number of 50 L bioreactor production per year			Number of 2000 L bioreactor production per year		
	Benchmark	USP optimized	USP Intensified	Benchmark	USP optimized	USP Intensified
RPE65 Mutation-associated Retinal Dystrophy	1	1	1	1	1	1
Age-related Macular Degeneration (AMD)	1667	114	107	42	3	3
Hemophilia B	10,326	704	661	228	18	17
Spinal Muscular Atrophy (SMA)	5072	346	324	127	9	8
Duchenne Muscular Dystrophy (DMD)	30,797	2100	1970	770	53	49
Hemophilia A	161,232	10,993	10,313	4031	275	258

C Cost per dose across the evaluated indications at 50 L and 2000 L production scales under benchmark, upstream-optimized (DOE1+ DOE2), and intensified USP scenarios.						
Indication	Cost ($)/dose for 50 L bioreactor scale			Cost ($)/dose for 2000 L bioreactor scale		
	Benchmark	USP optimized	USP Intensified	Benchmark	USP optimized	USP Intensified
RPE65 Mutation-associated Retinal Dystrophy	302	21	24	25	2	4
Age-related Macular Degeneration (AMD)	664	47	52	54	5	10
Hemophilia B	2,815,623	197,375	222,194	228,849	21,004	40,777
Spinal Muscular Atrophy (SMA)	3,418,970	236,670	269,807	277,888	25,505	49,515
Duchenne Muscular Dystrophy (DMD)	5,631,245	394,750	444,387	457,699	42,009	81,555
Hemophilia A	8,446,868	592,126	666,581	688,548	63,013	122,332

^1^The prevalence of each indication is referenced from [27, 30–33].

^2^The total number of patients worldwide was calculated based on [2].

^3^Refer to Table S10 (Supplementary Section) for details of total vg annual demand calculation.

^4^This probability applies exclusively to the male population, as these diseases are prevalent mainly among men. This is prevalence rather than birth incidence.

## Capsid enrichment and recovery trade-offs in AEX polishing

The economic impact of capsid enrichment during AEX polishing was evaluated under several scenarios for two molecules (Vector A and B), each reflecting progressively higher enrichment accompanied by corresponding losses in vector recovery. For full details of AEX optimization and corresponding results, refer to Supplementary Information (Section 6: Percent Full Capsid Enrichment via AEX). Enrichment is defined as the relative increase in full vector particles compared to each process’s load material. Reduced recovery was propagated through subsequent DSP operations, with costs recalculated to determine the total manufacturing cost and cost per 1×10^12^ vg. Additional process data supporting the model are provided in the Supplementary Information (Section 6).

**Fig. 9:**
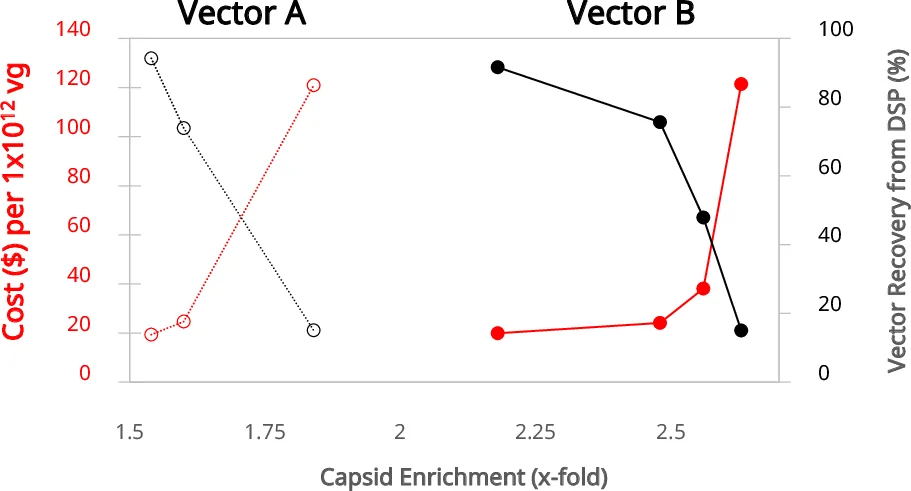
Trade-off between capsid enrichment, recovery, and cost efficiency in AEX polishing. Two vector molecules were compared across multiple enrichment scenarios, in which increased capsid purity was accompanied by corresponding losses in vector recovery. Although total downstream manufacturing costs remained approximately constant (~$4.2 M), reduced recoveries—from >90% at low enrichment to ~15% at maximum enrichment—caused the cost per 1 × 10¹² vg to increase nearly six-fold, from ~$20 to ~$120. Vector B achieved greater maximum enrichment than Vector A (2.63-fold vs. 1.84-fold) while maintaining similar step yields.

Total costs remained relatively stable across scenarios (approximately $4.2 M). However, as shown in Fig. 9, recovery decreases substantially with increasing enrichment. For Vector A, recovery declined from 94% at 1.6× enrichment to 16% at 1.84× enrichment. For Vector B, recovery dropped from 92% at 2.18× enrichment to 15% at 2.63× enrichment. The reduction in recovery with higher enrichment led to an approximately six-fold increase in cost per vg at the BDS stage (to $121.4 for Vector A and $120.9 for Vector B) at the maximum enrichment achieved for each vector. Notably, Vector B achieved a roughly 1.4-fold higher maximum enrichment than Vector A while maintaining comparable AEX step yields. This improvement was driven primarily by extensive process development efforts, including high-throughput screening for AEX optimization as outlined in the Supplementary Information (Table S9).

While higher enrichment improves capsid quality, the accompanying loss in yield disproportionately increases the cost per unit of product. Because overall process costs change little with enrichment, the cost per 1×10^12^ vg is primarily governed by recovery. These findings emphasize the central trade-off in AEX polishing: increasing enrichment can more than double product cost. The optimal operating point therefore likely lies at an intermediate enrichment level that balances product quality requirements with economic feasibility. While a trade-off between purity and yield exists, purity specifications are ultimately defined by safety and efficacy requirements and are not freely adjustable in late-stage development. Therefore, clinically relevant thresholds should be established during the preclinical and clinical stages, with process development optimized to meet these criteria.

## Indication-specific rAAV demand, batch requirements, and cost reduction

Table 1A summarizes global annual rAAV vg demand for the six leading indications considered suitable for rAAV gene therapy treatment: RPE65 mutation-associated retinal dystrophy, age-related macular degeneration (AMD), hemophilia A, hemophilia B, spinal muscular atrophy (SMA), and Duchenne muscular dystrophy (DMD). These indications span a broad range of dose requirements per patient, with retinal dystrophy requiring the lowest vg dose and Hemophilia A requiring the highest. Using the prevalence-based patient estimates and dosing assumptions outlined in the Supplementary Information (Section 7: Indication-Specific Patient Population, Dose, and rAAV Demand), the resulting annual worldwide vg demand spans more than six orders of magnitude—from 8.0 × 10^13^ vg for RPE65-associated retinal dystrophy to 7.6 × 10^19^ vg for Hemophilia A. Intermediate-range demands include 5.7×10^18^ vg for Hemophilia B, 2.8 × 10^18^ vg for SMA, and 1.7 × 10^19^ vg for DMD.

For the indications evaluated in this study, Table 1B summarizes the number of batches required to meet global annual demand as a function of vg requirements and production scale (50 L or 2000 L) under three scenarios: a benchmark rAAV production process with minimal optimization, a fully optimized transient-transfection process incorporating DOE1 + DOE2 improvements, and an intensified perfusion-based process. The vg titers at harvest were 6.0 × 10¹⁰, 8.8 × 10¹¹, and 9.4 × 10¹¹ vg/mL for the benchmark, optimized (DOE1 + DOE2), and intensified perfusion processes, respectively. Details of the optimization and intensification strategies are provided in the Supplementary Information (Sections 5 and 6).

Although fewer batches are needed at 2000 L than at 50 L, this number decreases further with process optimization and intensification for all indications. For clinical indications with low global vg demand (such as ultra-rare RPE65-associated retinal dystrophy), the benchmark process at 50 L—or even at smaller scales—is sufficient to supply the patient population. By contrast, indications with high annual demand in the low-to-mid 1×10¹⁹ vg range require not only large-scale batches (2000 L or greater) but also process optimization or intensification to maintain a manageable annual batch count (on the order of tens to hundreds of batches per year).

As shown in Table 1C, scale-up and process optimization or intensification each reduce cost per dose by approximately 6 ~ 12-fold. When combined, these effects result in an overall 70 ~ 150-fold reduction in cost per dose, reducing treatment costs from the million-dollar range to the low hundreds of thousands of dollars. Under such optimized and intensified conditions, the cost per dose for neuromuscular disorders, including SMA and DMD, can be reduced to approximately $50k–$80k. Process optimization and intensification are especially critical for neuromuscular indications such as SMA and DMD, which are increasingly important targets in the expanding gene therapy landscape for neuromuscular disease [1, 26, 27].

An important consideration not explicitly captured in this analysis is the potential transition from a prevalence-driven treatment phase to an incidence-driven demand profile. In the early stages of therapy adoption, manufacturing demand is dominated by treatment of the existing patient population, resulting in high annual vector requirements. However, once this prevalent population has been largely treated, demand is expected to decrease substantially and become primarily driven by newly diagnosed (incident) patients.

This shift has important implications for manufacturing strategy. Under an incidence-only scenario, reduced batch demand may lead to underutilization of large-scale facilities, thereby increasing the effective cost per dose due to fixed overhead and conversion costs. In such cases, smaller-scale, more flexible manufacturing platforms or modular production strategies may become more economically favorable compared to large-scale operations optimized for peak demand.

While the present study focuses on the prevalence-driven phase to evaluate maximum demand and capacity requirements, incorporating incidence-based demand profiles represents an important area for future work to further refine long-term manufacturing and capacity planning strategies.

A further limitation of this study is the use of a standardized cost framework, defined as a modeling approach based on publicly available data and industry benchmarks primarily derived from the United States and other developed biopharmaceutical markets, rather than a true global average. As such, the model does not capture regional differences in labor rates, facility costs, supply chain logistics, or regulatory environments. In practice, these factors can lead to substantial variation in COGs across manufacturing locations, particularly in emerging markets, where labor costs, supplier pricing, and infrastructure may differ substantially from those in US or European settings. While such variability was not explicitly modeled here, the framework presented could be extended to incorporate region-specific parameters in future analyses. Importantly, these regional effects are expected to primarily shift absolute cost levels, while the relative trends between platforms and process strategies observed in this study are likely to remain consistent.

## Conclusion

This study provides a holistic, cross-platform view of rAAV manufacturing economics that integrates indication-specific demand, production scale, platform choice, and process development strategy. Using the bottom-up cost model, we show that each platform has a distinct economic fingerprint: transient transfection is dominated by plasmids, transfection reagent, media, and endonuclease; PCL perfusion processes are driven by media and perfusion-related single-use hardware; and the baculovirus platform shifts a greater share of cost into downstream operations, particularly affinity capture. When normalized per 1 × 10¹² vg, cost rankings differ from those based on total batch cost, underscoring the need to interpret platform comparisons in the context of volumetric productivity and intended indication.

DOE–guided transfection optimization increased harvest titers by more than an order of magnitude and reduced cost per 1×10¹² vg from the low hundreds of dollars to the mid-teens, with corresponding multi-fold gains in patient coverage at fixed bioreactor scale. Converting a benchmark fed-batch process to a perfusion-intensified configuration increased titers by more than 50-fold and decreased cost per 1×10¹² vg from >$600 to <$30, even though absolute material costs rose substantially, highlighting the impact of upstream productivity for dose-intensive indications.

Downstream strategies provide complementary but more modest savings. Affinity resin reuse reduced the cost of the capture step by up to approximately 60%, but translated into only approximately 3–4% reductions in total material cost at the 2000 L scale, reflecting the growing dominance of upstream materials as processes intensify. Anion-exchange polishing revealed a critical trade-off: pushing capsid enrichment beyond approximately 1.5-fold increased the cost per 1×10¹² vg by more than two-fold, and in some cases up to six-fold, because of sharp declines in recovery despite essentially unchanged total batch cost. This finding emphasizes that DSP development must balance product quality improvements against the economic penalty of yield loss.

Incorporating indication-specific demand further reveals the practical consequences of process design. Benchmark 50 L processes are sufficient for ultra-rare diseases, whereas large indications with annual demand on the order of 10¹⁹ vg require optimized or intensified processes at 2000 L to maintain a feasible batch burden. In this model, scale-up combined with systematic process optimization or intensification reduced the estimated cost per dose by approximately 70- to 150-fold, lowering dose costs from the million-dollar range to the low hundreds of thousands of dollars. Under these modeled conditions, dose costs of approximately $50,000–$80,000 may be achievable for high-dose neuromuscular indications.

Overall, our analyses suggest there is no universally “best” rAAV platform. Optimal platform choice depends on indication prevalence, dose requirements, desired serotype flexibility, development timelines, and willingness to invest in process intensification. For small patient populations and rapidly evolving programs, transient transfection may remain the most pragmatic option despite higher per-batch material costs. In contrast, for larger indications requiring sustained supply, baculovirus infection and PCL platforms, combined with USP optimization and judicious downstream cost-control measures, may deliver superior long-term economics. Integrating quantitative cost modeling into platform selection and process development decisions early in program design will be essential for making rAAV therapies economically sustainable and expanding global patient access. Importantly, because gene therapy pricing is typically guided by value-based frameworks that align price with the magnitude of clinical benefit, including long-term survival gains and improvements in quality of life following a single administration, pricing may not be determined solely by manufacturing cost [5, 28].

## Supplementary information

rAAV Production Cost Analysis: Indication-Specific Cost per Dose and Reduction Strategies

## Data Availability

All data and supporting materials are included in the article and Supplementary Information. Raw data are available from the corresponding author upon reasonable request.
