# Supplementary material for: rAAV production cost analysis: Indication-specific cost per dose and reduction strategies
Source: Gene Ther. 2026 Jul 21;33(5):559–70. doi: 10.1038/s41434-026-00631-3 (PMC13585547; doi:10.1038/s41434-026-00631-3)
Supplement: Supplementary file 1 [file 41434_2026_631_MOESM1_ESM.docx]

**Supplementary Information**

**rAAV Production Cost Analysis: Indication-Specific Cost per Dose and Reduction Strategies**

Min Tae Park^[1],^^[2]^, Dan Matuszek^1,2^, Angela Andaluz^1^ and Seyed Pouria Motevalian^1, 🖂^

# ***1. Conversion Cost Model***

***Supplementary Table S1:*** *Conversion cost model equations (Adapted from Farid model* [1])[2, 3]

| **Cost category** | **Cost type** | **Equation** |
| --- | --- | --- |
| Labor cost  (per batch) | Operating labor (USP/DSP) | Number of operators × Annual salary ÷ (Batches/year) |
|  | DP/QC/QA labor | Number of operators × Annual salary ÷ (Batches/year) |
|  | Management labor^[3]^ | Number of workers × Annual salary ÷ (Batches/year) |
|  | Supervisors | Number of supervisors × Annual salary ÷ (Batches/year) |
| Indirect cost  (per batch) | Maintenance | 0.1 × FCI^[4]^ ÷ (Batches/year) |
|  | Local taxes | 0.2 × FCI ÷ (Batches/year) |
|  | Insurance | 0.01 × FCI ÷ (Batches/year) |
|  | Depreciation | FCI divided by depreciation period (years)^[5]^ ÷ (Batches/year) |
|  | General utilities | General utilities cost for rAAV manufacturing facility area ÷ (Batches/year) |

# ***2. PCL and BIICs Development Costs and Allocation to Manufacturing***

***Supplementary Table S2:*** *Information on outsourcing costs for cell line/virus creation, banking, etc.*

| **Platform** | **Item** | **Description** | **Price (k$)** | **Batches covered** |
| --- | --- | --- | --- | --- |
| **Sf9** | Cell bank construction | Cell expansion and banking of Sf9 cell | 120 | 300 – 400 |
|  | Cell bank characterization | To ensure safety and consistency of the cell bank (testing genetic ID/stability, sterility and physiological characteristics) | 90 |  |
|  | MBIICs manufacture | Generation of validated baculovirus-infected cells for seed virus bank | 1,913 | 27 |
|  | MBIICs characterization | To ensure safety (sterility) |  |  |
|  | **Cost (k$) per batch** | | **71.5** | |
|  | | | | |
| **PCL** | Cell line development | Construct a cell line complementing the virus gene or GOI in chromosome | 300 | 300 – 400 |
|  | Cell bank construction | Cell expansion and banking of PCL | 120 |  |
|  | Cell bank characterization | To ensure safety and consistency of the cell bank (testing genetic ID/stability, sterility and physiological characteristics) | 90 |  |
|  | License fee | Cost associated with obtaining the right to use proprietary technologies of developed cell line | 25 | Every batch |
|  | Virus development | To develop a helper virus having the assembly and packaging genes for the AAV vectors | 200 | 30 |
|  | Virus bank generation | Virus expansion and seed virus banking | 300 |  |
|  | Virus characterization | To ensure consistency of the virus bank | 90 |  |
|  | **Cost (k$) per batch** | | **46.1** | |

# ***3. Downstream Processing - Steps and Recoveries***

**Supplementary Table S3:** Purification step recoveries [4].

| **Process** | **Recovery (%)** |
| --- | --- |
| Clarification | 70.0 |
| hTFF^[6]^ | 83.5 |
| Affinity | 75.5 |
| AEX^[7]^ | 63.5 |
| VRF^[8]^ | 100 |
| fTFF^[9]^ | 82.0 |
| Overall recovery | 23.0 |

# ***4. Transfection Process Optimization***

## 4.1 Materials and Methods

### 4.1.1. Seed train

A continuous seed train was maintained for all experiments by thawing a vial of HEK293F Viral Production Cells (Gibco, Waltham, MA, USA) into 30 mL of LV-MAX Production Medium (Gibco, Waltham, MA, USA) and culturing the cells for 4 days in a 125-mL shake flask (Corning, Corning, NY, USA). After thaw, the seed train was expanded by inoculating 125-mL to 2-L shake flasks at 40% working volume with target seeding densities of 3.0×10⁵ cells/mL for 4-day passages and 5.0×10⁵ cells/mL for 3-day passages. All cultures were maintained in a 37 °C, 5% CO₂ incubator at 125 rpm. The seed train was discarded after 20 passages, at which point a new vial was thawed to restart the process.

### 4.1.2. Bioreactor parameters

The ambr®15 (48-vessel) cell culture system (Sartorius, Göttingen, Germany) was inoculated at half of the target transfection cell density for both Design-of-Experiments (DOEs) and cultured for one day prior to transfection. Cultures were maintained at a pH setpoint of 7.0 ± 0.2, a dissolved oxygen (DO) setpoint of 30%, an air sparge rate of 0.2 mL/min, and an agitation speed of 820 rpm. Following transfection, the pH setpoint was adjusted to 6.9 ± 0.1.

### 4.1.3. Production optimization

DOE1 evaluated the effects of viable cell density, total DNA, and transfection reagent-to-DNA ratio at transfection, following the experimental design outlined in Table S4. DOE2 assessed optimal plasmid ratios using the conditions summarized in Table S5. For both studies, FectoVIR-AAV transfection reagent (Sartorius, Göttingen, Germany), a 30-min complexation time, 18.9-kb PALD-X80 helper plasmid (Aldevron, Fargo, ND, USA), and a custom 5.2-kb pGOI plasmid (Aldevron, Fargo, ND, USA) were used. RepCap plasmid consisted of a 6.1-kb pALD construct (Aldevron, Fargo, ND, USA). DOE1 employed a fixed plasmid ratio of 0.15:0.43:0.42 (pGOI:pRC:pHelper), whereas DOE2 incorporated the optimal parameters identified in DOE1. JMP statistical software (JMP Group, San Francisco, CA, USA) was used to design and analyze the DOEs using a response surface methodology approach.

All cultures were harvested 3 days post-transfection. Lysis was performed by treating each culture with 30 mM Tris pH 8.0 (Gibco, Waltham, MA, USA), 2 mM MgCl₂ (Gibco, Waltham, MA, USA), 150 mM NaCl (Gibco, Waltham, MA, USA), 0.2% Tween-20 (Sigma-Aldrich, Burlington, MA, USA), and 90 U/mL Benzonase (Sigma-Aldrich, Burlington, MA, USA) for 2 hours.

***Supplementary Table S4:*** *Experimental design for DOE1*

| **Run number** | **VCD (×10^6^ cells/mL)** | **Total DNA (µg per 10^6^ cells)** | **FectoVIR-to-DNA ratio** |
| --- | --- | --- | --- |
| 1 | 4.0 | 0.4 | 0.4 |
| 2 | 4.0 | 1 | 0.4 |
| 3 | 4.3 | 0.7 | 0.8 |
| 4 | 3.0 | 0.7 | 1.31 |
| 5 | 2.0 | 0.4 | 1.2 |
| 6 | 2.0 | 0.4 | 0.4 |
| 7 | 2.0 | 1 | 0.4 |
| 8 | 2.0 | 1 | 1.2 |
| 9 | 4.0 | 1 | 1.2 |
| 10 | 3.0 | 0.314 | 0.8 |
| 11 | 1.7 | 0.7 | 0.8 |
| 12 | 3.0 | 1.086 | 0.8 |
| 13 | 3.0 | 0.7 | 0.8 |
| 14 | 4.0 | 0.4 | 1.2 |
| 15 | 2.4 | 0.25 | 1 |
| 16 | 3.0 | 0.7 | 0.29 |
| 17 | 3.0 | 0.5 | 1 |
| 18 | 3.0 | 0.25 | 1 |
| 19 | 4.0 | 0.25 | 1 |
| 20 | 2.0 | 1 | 1 |
| 21 | 3.0 | 1 | 1 |
| 22 | 4.0 | 1 | 1 |
| 23 | 2.0 | 0.25 | 1 |
| 24 | 3.0 | 0.7 | 0.8 |
| 25 | 4.0 | 0.4 | 0.4 |
| 26 | 4.0 | 1 | 0.4 |
| 27 | 4.3 | 0.7 | 0.8 |
| 28 | 3.0 | 0.7 | 1.31 |
| 29 | 2.0 | 0.4 | 1.2 |
| 30 | 2.0 | 0.4 | 0.4 |
| 31 | 2.0 | 1 | 0.4 |
| 32 | 2.0 | 1 | 1.2 |
| 33 | 4.0 | 1 | 1.2 |
| 34 | 3.0 | 0.314 | 0.8 |
| 35 | 1.7 | 0.7 | 0.8 |
| 36 | 3.0 | 1.086 | 0.8 |
| 37 | 3.0 | 0.7 | 0.8 |
| 38 | 4.0 | 0.4 | 1.2 |
| 39 | 2.4 | 0.25 | 1 |
| 40 | 3.0 | 0.7 | 0.29 |
| 41 | 3.0 | 0.5 | 0.8 |
| 42 | 3.0 | 0.25 | 1 |
| 43 | 4.0 | 0.25 | 1 |
| 44 | 2.0 | 1 | 1 |
| 45 | 3.0 | 1 | 1 |
| 46 | 4.0 | 1 | 1 |
| 47 | 2.0 | 0.25 | 1 |
| 48 | 3.0 | 0.7 | 0.8 |

***Supplementary Table S5:*** *Experimental design for DOE2*

| **Run number** | **pGOI mass ratio** | **pRep/Cap mass ratio** | **pHelper mass ratio** |
| --- | --- | --- | --- |
| 1 | 0.05 | 0.37 | 0.58 |
| 2 | 0.07 | 0.20 | 0.73 |
| 3 | 0.30 | 0.20 | 0.50 |
| 4 | 0.72 | 0.14 | 0.14 |
| 5 | 0.33 | 0.05 | 0.62 |
| 6 | 0.22 | 0.56 | 0.21 |
| 7 | 0.88 | 0.06 | 0.07 |
| 8 | 0.21 | 0.12 | 0.67 |
| 9 | 0.11 | 0.07 | 0.82 |
| 10 | 0.50 | 0.27 | 0.23 |
| 11 | 0.07 | 0.61 | 0.33 |
| 12 | 0.15 | 0.43 | 0.42 |
| 13 | 0.60 | 0.06 | 0.34 |
| 14 | 0.18 | 0.29 | 0.52 |
| 15 | 0.06 | 0.87 | 0.07 |
| 16 | 0.10 | 0.72 | 0.18 |
| 17 | 0.43 | 0.09 | 0.47 |
| 18 | 0.27 | 0.35 | 0.38 |
| 19 | 0.39 | 0.52 | 0.10 |
| 20 | 0.28 | 0.66 | 0.06 |
| 21 | 0.54 | 0.17 | 0.29 |
| 22 | 0.45 | 0.39 | 0.16 |
| 23 | 0.64 | 0.31 | 0.05 |
| 24 | 0.32 | 0.43 | 0.25 |

## 4.2 Analytical testing

For viral genome (vg) titer quantification, samples were treated with DNase I (New England BioLabs, Ipswich, MA, USA) and Proteinase K (Thermo Fisher Scientific, Waltham, MA, USA), followed by serial dilution. Droplet digital PCR (ddPCR) was performed using the QX200 Auto DG Droplet Digital PCR System (Bio-Rad, Hercules, CA, USA), targeting the vector promoter sequence to determine vg titer. Capsid particle concentrations were quantified using AAV Capsid ELISA kits (Kactus Bio, Shanghai, China).

## 4.3 Results

Vg titers following DOE1 and combined DOE1+DOE2 optimizations were 2.9×10^11^ vg/mL and 8.8×10^11^ vg/mL, respectively, representing 4.8-fold and 14.6-fold increases in productivity relative to the benchmark titer (6×10¹⁰ vg/mL) [1]. The optimal parameters identified included a viable cell density at transfection of 3×10⁶ cells/mL, a total DNA usage of 0.6 µg per 10⁶ cells, and a FectoVIR-to-DNA ratio of 1.3.

# ***5. Upstream Process Intensification via Perfusion***

## 5.1 Materials and Methods

### 5.1.1. Seed train

Viral Production Cells 2.0 (Thermo Fisher Scientific, Waltham, MA, USA), derived from HEK293 cells, were thawed and expanded in flat-bottom, vented shake flasks at 20–40% working volume using LV-MAX™ Production Medium (Thermo Fisher Scientific, Waltham, MA, USA). Cells were passaged every 3 or 4 days at seeding densities of 0.6×10⁶ cells/mL or 0.4×10⁶ cells/mL, respectively, and cultured at 37 °C, 125 rpm, and 5% CO₂ on a 25-mm orbital-throw shaker (Thermo Fisher Scientific, Waltham, MA, USA). Viable cell density was measured at each passage using a Vi-CELL XR (Beckman Coulter, Brea, CA, USA).

### 5.1.2. Production bioreactor, perfusion and harvest

ambr®250HT cell culture system (Sartorius, Göttingen, Germany) was used for perfusion. Culture stations were maintained at 37 °C with pH controlled between 6.8 and 7.2. pH regulated using 1 M sodium carbonate for base addition and CO₂ sparging for acidification. Dissolved oxygen (DO) was maintained at 30% using a constant air sparge with supplemental O₂ as needed. Daily samples were collected from all bioreactors to assess metabolites and offline gases using a Vi-CELL MetaFLEX (Beckman Coulter, Brea, CA, USA), and to measure viable cell density and viability using a Vi-CELL XR.

The ambr®250HT bioreactor system was operated using Perfusion TFF vessels equipped with 0.2-µm filters. Each bioreactor was charged with 125 mL of medium, and TFF columns were wetted using a crossflow rate of 45 mL/min. Perfusion was initiated at 0.5 vessel volume per day (VVD) on Day 1 and subsequently increased to 0.9, 1.5, and 1.8 VVD over the next three days. Perfusion was halted immediately prior to transfection. For fed-batch processes, perfusion remained off following transfection. For vessels employing post-transfection perfusion, perfusion was resumed approximately 4.5 hours after completion of the transfection.

Cells were transfected to final working viable cell densities of 3.5×10^6^, 10.5×10^6^ and 14.0×10^6^ cells/mL using identical DNA and FectoVIR-AAV transfection reagent amounts per 10⁶ cells for each respective process. Transfection mixes were linearly scaled to maintain equivalent concentrations of DNA and transfection reagent per 10⁶ cells. Carrier medium (LV-MAX) was also scaled proportionally, using 10% complexation for each fold increase in viable cell density.

During perfusion, cells were further concentrated prior to transfection to ensure that the post-transfection working volume and target cell density were achieved after adding the transfection mixture. Transfection mixes were made using 0.43 µg of DNA per 10⁶ cells, with a plasmid mass ratio of 0.283:0.263:0.453 (GOI:Rep/Cap:Helper). Plasmid DNA was first added to the carrier medium, followed by FectoVIR-AAV, and the mixture was gently mixed to ensure homogeneity. Complexes were incubated for 30 minutes before being added to cultures by pipetting.

Approximately 72 hours post-transfection, DO and pH control were turned off, and the culture was treated with lysis buffer components to reach final concentrations of 0.2 % Polysorbate-20 (Sigma-Aldrich, Burlington, MA), 2 mM MgCl₂ (Sigma-Aldrich, Burlington, MA), 50 U/mL Benzonase (Sigma-Aldrich, Burlington, MA), and 150 mM NaCl (Sigma-Aldrich, Burlington, MA). The culture was incubated for 120 minutes. Samples were then collected from the bioreactor, centrifuged at 2000 ×g for 10 minutes, and the clarified supernatant was harvested for analytical testing.

## 5.2 Analytical testing

For vg titer quantification, samples were treated with DNase I (New England BioLabs, Ipswich, MA, USA) and Proteinase K (Thermo Fisher Scientific, Waltham, MA, USA), followed by serial dilution. Droplet digital PCR (ddPCR) was performed using the QX200 Auto DG Droplet Digital PCR System (Bio-Rad, Hercules, CA, USA), targeting the vector promoter sequence to determine vg titer. Capsid particle concentrations were quantified using AAV Capsid ELISA kits (Kactus Bio, Shanghai, China).

## 5.3 Results

Vg titer for the perfusion pre-transfection and pre-/post-transfection processes were 3.4×10^11^ vg/mL and 9.4×10^11^ vg/mL, respectively, representing 5.7-fold and 15.7-fold increases relative to the industrial benchmark (6×10^10^ vg/mL).

# ***6. Percent Full Capsid Enrichment via AEX***

## 6.1 Materials and Methods

### 6.1.1. AEX chromatography runs

Affinity-purified AAV samples (serotype 9 for Vector A and serotype 5 for Vector B) were subjected to separate AEX development workflows aimed at maximizing full-capsid enrichment. For Vector B, development was conducted using a high-throughput screening approach. Sixteen individual elution conditions were evaluated, including variations in buffer composition and ionic strength, followed by combinatorial pooling of elution fractions. Screening experiments were performed on a Tecan Freedom EVO 1080 (Tecan, Männedorf, Switzerland) liquid-handling system using POROS 50HQ RoboColumns (Thermo Fisher Scientific, Waltham, MA, USA). For Vector A, three lower-resolution, at-scale screening runs were conducted to evaluate the effect of different elution buffer sodium chloride concentrations on capsid enrichment and recovery over a Mustang Q column (Cytiva, Marlborough, MA, USA).

### 6.1.2. Vector B (AAV5) high throughput screening elution optimization

A custom Tecan script was developed to enable testing of up to eight different elution conditions in parallel using a pseudo-linear gradient approach across eight RoboColumns. Each pseudo-gradient step was applied over one column volume. The ionic strength profile of each elution buffer varied depending on its specific formulation and constituent salts.

Elution fractions were monitored by UV absorbance at 260 nm and 280 nm to identify primary elution peaks. Individual fractions were subsequently analyzed for vector genome titer by ddPCR and the ratio of empty to full capsids using mass photometry. Mock elution pools were generated to determine which of the sixteen total screening conditions produced the highest full-vector concentration. Multiple permutations of the optimal elution pool were then evaluated to illustrate the trade-off between capsid enrichment and vector recovery within a well-characterized AEX process. The raw data from each individual fraction of the selected conditions is shown in Table S6 while data extrapolated from the mock pools are shown in Table S7.

***Supplementary* Table *S6*:** *Vg titer, total vg, vector recovery, percent full capsids, and estimated total capsid concentrations for each elution fraction collected across the eight pseudo-linear gradient conditions evaluated during high-throughput AEX process development*.

| **Sample** | **Vg titer (vg/mL)** | **Total vg** | **Vg recovery (%)** | **% Full** | **Total capsid estimations** |
| --- | --- | --- | --- | --- | --- |
| Load | 2.38×10^13^ | 4.76×10^12^ | N/A | 31.9 | 1.49×10^13^ |
| Elution CV A | 3.80×10^12^ | 7.60×10^11^ | 16 | 45.1 | 1.69×10^12^ |
| Elution CV B | 6.62×10^12^ | 1.32×10^12^ | 28 | 75.4 | 1.75×10^12^ |
| Elution CV C | 6.34×10^12^ | 1.27×10^12^ | 27 | 80.9 | 1.57×10^12^ |
| Elution CV D | 3.58×10^12^ | 7.16×10^11^ | 15 | 83.9 | 8.53×10^11^ |
| Elution CV E | 1.47×10^12^ | 2.94×10^11^ | 6 | 79.6 | 3.69×10^11^ |

***Supplementary* Table *S7*:** *Theoretical vg yield, percent full capsids, and calculated capsid enrichment levels for each mock elution pool generated from the high-throughput AEX screening study.* *These pooled datasets were used to compare the trade-off between vector recovery and enrichment across the evaluated elution strategies*.

| **Elution pool combination** | **% Vg yield** | **% Full** | **Enrichment** |
| --- | --- | --- | --- |
| Elution CV D | 15.0 | 83.9 | 2.63 |
| Elution CVs D +C | 41.7 | 82.0 | 2.57 |
| Elution CVs D +C +E | 47.9 | 81.6 | 2.56 |
| Elution CVs D +C +E+ B | 75.6 | 79.2 | 2.48 |
| Elution CVs D +C +E+ B +A | 91.6 | 70.0 | 2.19 |

### 6.1.3. Vector A (AAV9) simple elution buffer screening

Several small-scale scouting runs were performed on a Mustang Q membrane column using an ÄKTA Avant 25 (Cytiva, Marlborough, MA, USA) system. Each run employed elution buffers with slightly different constituent concentrations, applied through a traditional linear gradient. The three elution fractions with the highest UV 260:280 absorbance ratios were pooled and evaluated for vector recovery by ddPCR and percent full capsids by mass photometry. A total of six runs were performed, of which three produced desirable results for further evaluation. Recovery and enrichment data from each of the pooled elution peaks are shown in Table S8.

***Supplementary* Table *S8*:** *Vector recovery and capsid enrichment results for pooled elution peaks obtained from six small-scale Mustang Q scouting runs on the ÄKTA Avant 25 system*.

| **Sample** | **Total vg** | **Vg recovery (%)** | **% Full** | **Enrichment** |
| --- | --- | --- | --- | --- |
| Load condition 1 | 1.80×10^13^ |  | 35 |  |
| Mustang Q elution peak cond 1 | 2.53×10^12^ | 14 | 64 | 1.84 |
| Load condition 4 | 1.79×10^13^ |  | 35 |  |
| Mustang Q elution peak cond 4 | 1.32×10^13^ | 74 | 55 | 1.60 |
| Load condition 6 | 1.69×10^13^ |  | 35 |  |
| Mustang Q elution peak cond 6 | 1.59×10^13^ | 94 | 53 | 1.54 |

## 6.2 Analytical testing

Empty and full AAV capsid ratios were determined using a SamuxMP Auto mass photometer (Refeyn, Oxford, UK), which measures single-particle light scattering proportional to molecular mass. Samples were diluted in appropriate buffers and loaded into 96-well plates for automated acquisition using AcquireMP software. Automated dilutions ensured valid measurements within a target range of 1,500–3,000 binding counts.

Calibration was performed using the MassFerence P2 standard (Refeyn, Oxford, UK) to convert contrast values to molecular mass. Each measurement was acquired over 1 minute following auto-focus adjustment under default laser settings. Data were processed using DiscoverMP software to quantify the proportion of empty, partially filled, and full capsids. All samples were analyzed in technical replicates to confirm reproducibility.

Vg titers were quantified using the QX200 AutoDG Droplet Digital PCR System (Bio-Rad, Hercules, CA, USA). Samples were treated sequentially with DNase I (New England Biolabs, Ipswich, MA, USA) to remove residual plasmid DNA and Proteinase K (Thermo Fisher Scientific, Waltham, MA, USA) to release encapsidated genomes, followed by serial dilution to ensure measurements within the assay’s dynamic range.

Amplification was performed using ddPCR Supermix for Probes (No dUTP) (Bio-Rad) with custom probe and primer sets specific to the gene of interest. Droplet generation, PCR cycling, and fluorescence detection were carried out per manufacturer instructions, and vg titers were reported as copies per milliliter based on droplet partitioning statistics.

## 6.3. Results

The influence of varying enrichment levels on vector recovery and corresponding process costs is presented in Supplementary Table S9. Recovery adjustments derived from the AEX experiments for Vector A and Vector B were integrated into the overall process recovery model, and the resulting cost impacts were propagated through both process- and raw material–level cost structures. All outputs were normalized to report the modified cost per 1×10¹² vg across the evaluated scenarios.

Across all modeled conditions, yield decreased as capsid enrichment increased, consistent with expectations for AEX-based separation. However, the application of high-throughput process development enabled the identification of conditions that maintained comparable vector recoveries while achieving markedly improved enrichment.

***Supplementary* Table *S9*:** *Summary of modeled vector recoveries and associated manufacturing costs following integration of AEX-derived recovery adjustments for Vector A and Vector B. Cost impacts were propagated through process- and raw material–level models and normalized to reflect the modified cost per 1×10¹² vg for each enrichment scenario.*

| **Process attributes** | **Vector A** | | | | **Vector B** | | |
| --- | --- | --- | --- | --- | --- | --- | --- |
| Full particle enrichment | 2.19 | 2.48 | 2.56 | 2.63 | 1.54 | 1.6 | 1.84 |
| Modified vector step recovery (%) | 91.6 | 75.6 | 47.9 | 15.0 | 14.1 | 73.9 | 94.2 |
| Total modified manufacturing costs ($)^a^ | 4,229,155 | 4,228,192 | 4,226,519 | 4,224,537 | 4,229,312 | 4,228,087 | 4,224,541 |
| Modified DS recovery (%) | 33.1 | 27.4 | 17.3 | 5.4 | 34.1 | 26.7 | 5.5 |
| Modified total vg from BDS (vg)^[10]^ | 2.12×10^17^ | 1.75×10^17^ | 1.11×10^17^ | 3.48×10^16^ | 2.18×10^17^ | 1.71×10^17^ | 3.49×10^16^ |
| Modified cost per 1x10^12^ vg ($) | 19.95 | 24.16 | 38.13 | 121.38 | 19.40 | 24.72 | 120.90 |

# ***7. Indication-Specific Patient Population, Dose, and rAAV Demand ^[11]^***

***Supplementary Table S10:*** *Breakdown of eligible patient numbers, dose per patient, and corresponding global and annual vg demand for major indications treated with rAAV-based gene therapies.* [5-9]

*1. RPE65 mutation-associated retinal dystrophy*

| **Region** | **Number of patients** | **Diagnostic rates and access to genetic testing (%)** | **Clinical eligibility criteria (%)** | **Eligible patients** | **Dose (vg)/patient** | **Global demand (vg) for patients** | **Yearly global vg demand ^[12]^** |
| --- | --- | --- | --- | --- | --- | --- | --- |
| **US+Europe** | 2,730 | 50 | 80 | 1,092 | 1.5×10^11^ | 1.6×10^14^ | **8.0×10^13^** |
| **Asia** | 12,088 | 30 |  | 2,901 |  | 4.4×10^14^ |  |
| **ROW ^[13]^** | 5,796 | 20 |  | 927 |  | 1.4×10^14^ |  |
| **Total** | 20,614 |  |  | 4,921 |  | **7.4×10^14^** |  |

Prevalence: 0.25 per 100k

*2. Age-related macular degeneration (AMD)*

| **Region** | **Number of patients** | **Distribution (%)** | **Clinical eligibility criteria (%)** | **Eligible patients** | **Dose (vg)/patient** | **Global demand (vg) for patients** | **Yearly global vg demand ^b^** |
| --- | --- | --- | --- | --- | --- | --- | --- |
| **US+Europe** | 288,000,000 ^[14]^ | 39 | 50 | 28,080,000 | 3.3×10^11^ ^[15]^ | 9.3×10^18^ | **9.2×10^17^ ^[16]^** |
| **Asia** |  | 48 | 30 | 12,441,600 |  | 4.1×10^18^ |  |
| **ROW ^c^** |  | 13 | 20 | 1,497,600 |  | 4.9×10^17^ |  |
| **Total** |  |  |  | 42,019,200 |  | **1.4×10^19^** |  |

Prevalence: 350 per 100k

*3. Spinal muscular atrophy (SMA)*

| **Region** | **Number of patients** | **Diagnostic rates and access to genetic testing (%) [****10****]** | **Clinical eligibility criteria (%)** | **Eligible patients** | **Dose (vg)/patient** | **Global demand (vg) for patients** | **Yearly global vg demand** |
| --- | --- | --- | --- | --- | --- | --- | --- |
| **US+Europe** | 4,914 | 50 | 80 | 1,966 | 1.7×10^15^ | 3.3×10^18^ | **2.8×10^18^** |
| **Asia** | 21,759 | 30 |  | 5,222 |  | 8.9×10^18^ |  |
| **ROW** | 10,432 | 20 |  | 1,669 |  | 2.8×10^18^ |  |
| **Total** | 37,105 |  |  | 8,857 |  | **1.5×10^19^** |  |

Prevalence: 1 per 100k

*4. Hemophilia B*

| **Region** | **Number of patients** | **Diagnostic rates and access to genetic testing (%)** | **Clinical eligibility criteria (%) [****11-13****]** | **Eligible patients** | **Dose (vg)/patient** | **Global demand (vg) for patients** | **Yearly global vg demand** |
| --- | --- | --- | --- | --- | --- | --- | --- |
| **US+Europe** | 20,749 | 90 | 60 | 11,204 | 1.4×10^15^ | 1.6×10^19^ | **5.7×10^18^** |
| **Asia** | 91,871 | 40 |  | 22,049 |  | 3.1×10^19^ |  |
| **ROW** | 44,046 | 15 |  | 3,964 |  | 5.6×10^19^ |  |
| **Total** | 156,666 |  |  | 37,217 |  | **5.2×10^19^** |  |

Prevalence: 3.8 per 100k ^[17]^

*5. Hemophilia A*

| **Region** | **Number of patients** | **Diagnostic rates and access to genetic testing (%)** | **Clinical eligibility criteria (%)** | **Eligible patients** | **Dose (vg)/patient** | **Global demand (vg) for patients** | **Yearly global vg demand** |
| --- | --- | --- | --- | --- | --- | --- | --- |
| **US+Europe** | 93,369 | 90 | 60 | 50,419 | 4.2×10^15^ | 2.1×10^20^ | **7.6×10^19^** |
| **Asia** | 413,420 | 40 |  | 99,221 |  | 4.2×10^20^ |  |
| **ROW** | 198,208 | 15 |  | 17,839 |  | 7.5×10^19^ |  |
| **Total** | 704,996 |  |  | 167,479 |  | 7.0×10^20^ |  |

Prevalence: 17.1 per 100k ^a^

*6. Duchenne muscular dystrophy (DMD)*

| **Region** | **Number of patients** | **Diagnostic rates and access to genetic testing (%)** | **Clinical eligibility criteria (%)** | **Eligible patients** | **Dose (vg)/patient** | **Global demand (vg) for patients** | **Yearly global vg demand** |
| --- | --- | --- | --- | --- | --- | --- | --- |
| **US+Europe** | 38,767 | 70 | 60 | 16,282 | 2.8×10^15^ | 4.6×10^20^ | **1.7×10^19^** |
| **Asia** | 171,654 | 35 | 55 | 33,043 |  | 9.3×10^20^ |  |
| **ROW** | 82,297 | 15 | 45 | 5,555 |  | 1.6×10^19^ |  |
| **Total** | 292,718 |  |  | 54,881 |  | 1.5×10^20^ |  |

Prevalence: 7.1 per 100k ^[18]^

# ***8. Sensitivity Analysis of Total Cost to Material Cost and Productivity Assumptions***

Sensitivity analyses were performed to evaluate the impact of uncertainty and variability in key cost model inputs—such as raw material costs and upstream productivity—on the resulting cost per 1×10¹² vg, assuming transient transfection at 2000 L. Specifically, each input parameter was independently varied by ±10% while holding all others constant to isolate its individual effect on total manufacturing cost. The resulting changes were then compared to the baseline scenario to identify the most influential cost drivers.

As shown in Supplementary Fig. S1, upstream cost drivers—particularly plasmid DNA, media, and transfection reagents—have the greatest impact on total cost. Notably, a 10% change in plasmid DNA cost corresponds to approximately a 2% change in total cost per 1×10¹² vg, whereas most downstream components contribute comparatively minor effects. Supplementary Fig. S2A further illustrates the impact of ±10% variation in titer on cost per 1×10¹² vg. As shown, a 10% increase in titer results in an approximately 9% reduction in cost. This analysis illustrates the strong dependence of cost per dose on upstream titer assumptions and provides context for evaluating platform-specific productivity scenarios.

Given ongoing advancements in the PCL platform, higher productivity is anticipated in the coming years. Accordingly, the model was used to predict cost per 1×10¹² vg following 2x, 5x, and 10x improvements in upstream PCL titer, with results summarized in Supplementary Fig. S2B. The cost per 1×10¹² vg decreases proportionally with increased productivity up to 10x. Notably, a 1-log improvement in PCL titer is predicted to result in an approximately 10-fold reduction in cost per 1×10¹² vg. These results also suggest that improvements in PCL productivity could substantially enhance its economic competitiveness, reinforcing the importance of ongoing process development in this platform.

**References**

1.	Lyle A, Stamatis C, Linke T, Hulley M, Schmelzer A, Turner R, et al. Process economics evaluation and optimization of adeno‐associated virus downstream processing. Biotechnol Bioeng. 2024;121:2435-2448.

2.	Duke Finance. GAP 200.090, plant & equipment depreciation. 2025; Available from: <https://finance.duke.edu/accounting/gap/m200-090/>.

3.	Bafaqih M. Fixed asset useful life table: a comprehensive guide. 2025; Available from: <https://cpcongroup.com/insights/article/fixed-asset-useful-life-table/>.

4.	Park MT, Verma A, Froelich CA, Motevalian SP. Process and quality considerations for recombinant adeno-associated virus manufacturing platforms. Trends Biotechnol. 2025;43:1921-1937.

5.	Worldometers. World Population - Worldometers. 2025; Available from: <https://www.worldometers.info/world-population/>.

6.	Wikipedia Contributors. Population growth — Wikipedia. 2025; Available from: <https://en.wikipedia.org/wiki/Population_growth>.

7.	Wong WL, Su X, Li X, Cheung CMG, Klein R, Cheng C-Y, et al. Global prevalence of age-related macular degeneration and disease burden projection for 2020 and 2040: a systematic review and meta-analysis. The Lancet Glob Heal. 2014;2:e106-e116.

8.	GenSight Biologics. GenSight Biologics announces 1 Year safety data and efficacy signals from PIONEER Phase I/II clinical trial of GS030, an optogenetic treatment candidate for retinitis pigmentosa. 2023; Available from: <https://www.gensight-biologics.com/2023/02/13/gensight-biologics-announces-1-year-safety-data-and-efficacy-signals-from-pioneer-phase-i-ii-clinical-trial-of-gs030-an-optogenetic-treatment-candidate-for-retinitis-pigmentosa/?utm_source=chatgpt.com>.

9.	Kim S, Shim J, Bak J, Yi S, Han JS. Low-dose AAV gene therapy NG101 for wet AMD: preclinical results and ongoing phase 1/2a trial design. IOVS. 2025;66:2471-2471.

10.	Vrščaj E, Dangouloff T, Osredkar D, Servais L, Group SNWS. Newborn screening programs for spinal muscular atrophy worldwide in 2023. J Neuromuscul Dis. 2024;11:1180-1189.

11.	Alshehri A, Dougherty JA, Beckman L, Svensson M. A systematic review of cost-effectiveness analyses of gene therapy for hemophilia type A and B. J Manag Care Spec Pharm. 2024;30:1178-1188.

12.	O'Mahony B, Black C. Expanding hemophilia care in developing countries. Semin Thromb Hemost, 2005. Thieme Medical Publishers, 561-568.

13.	Zhu L, Zheng C. Interpretation of the “2022 World Hemophilia Annual Report” by the world federation of hemophilia. J Diagn Concepts Pract. 2024;23:40-45.

**Supplementary Fig. S1:** Sensitivity of total manufacturing cost to ±10% variation in individual material cost components

**Supplementary Fig. S2:** (A) Sensitivity of cost per 1*×*10¹² vg to ±10% variation in upstream productivity (vg titer). (B) Reduction in cost per 1*×*10¹² vg as a function of titer improvement in the PCL platform.

1. BioProcess Sciences, Pharma Services, Viral Vector Services, Thermo Fisher Scientific, Plainville MA 02762, USA
2. Both authors contributed equally to this work

   ^🖂^ Correspondence: [pouria.motevalian@thermofisher.com](mailto:pouria.motevalian@thermofisher.com) (S. P. Motevalian)
3. Management departments include project management, MSAT, engineering, facility, automation, validation, procurement, warehouse, etc.
4. FCI: Fixed Capital Investment. Costs are only for the rAAV manufacturing-related facility.
5. Depreciation for a GMP manufacturing plant is the systematic allocation of the cost of the facility's buildings and structures over 15 to 20 years [2, 3].
6. Harvest Tangential Flow Filtration
7. Anion-EXchange chromatography
8. Viral Reductive Filtration
9. Final Tangential Flow Filtration
10. Modified total vg from bulk drug substance (BDS) and the corresponding adjusted manufacturing costs were calculated relative to the total vg yield from a 2000 L–scale production run, modeled after the previously described transient transfection process
11. Assuming the total population of the world is 8.2 billion [5]
12. Treatment rationale and 10-year outlook: all existing patients treated over 10 years, plus newborns treated annually (assuming 70M people↑/year with net increase rate [5, 6].
13. Rest of World
14. As of 2020, the global number of AMD patients is approximately 196 million. This number is expected to increase to about 288 million by 2040 [7].
15. For AMD AAV gene therapy, doses reported across different approaches ranged from approximately 3×10⁹ to 5×10¹¹ vg per eye; for analysis, we considered the average value of about 3.3×10¹¹ vg per eye [8, 9].
16. Annual vg requirement calculated under the assumption of treating all AMD patients by 2040
17. This probability applies exclusively to the male population, as these diseases are prevalent mainly among men.
18. This probability applies exclusively to the male population, as these diseases are prevalent mainly among men
